# Redox and physiological responses of wheat to wheat streak mosaic virus under liposomal bionanocomposite seed treatment

**DOI:** 10.1007/s00253-026-13821-1

**Published:** 2026-04-17

**Authors:** Tetiana Nyzhnyk, Oleksiy Kovalenko, Halyna Snihur, Dmytro Kiriziy, Anhelina Kyrychenko

**Affiliations:** 1 Department of Symbiotic Nitrogen Fixation, Institute of Plant Physiology and Genetics of the National Academy of Sciences of Ukraine, Vasylkivska 31/17, Kiev, 03022 Ukraine; 2 Plant Virology Laboratory, D.K. Zabolotny Institute of Microbiology and Virology of the National Academy of Sciences of Ukraine, Acad. Zabolotny Str, Kiev, D03680 Ukraine; 3 Virology Department, ESC “Institute of Biology and Medicine of Taras Shevchenko National University of Kyiv, 64/13 Volodymyrska Str, Kiev, 01601 Ukraine; 4 Laboratory of Plant Pathogenesis, Institute of Biochemistry and Biophysics of the Polish Academy of Sciences, Adolfa Pawinskiego 5A, Warsaw, 02-106 Poland

**Keywords:** Antioxidant enzymes, Liposomal bionanocomposites, Plant resistance, Redox homeostasis, Wheat streak mosaic virus

## Abstract

**Abstract:**

Wheat streak mosaic virus (WSMV) is a serious viral pathogen of wheat and a major threat to global food security. Infection of susceptible plants triggers oxidative stress and disrupts physiological and biochemical homeostasis, resulting in substantial yield losses. Since chemical control of viral diseases is ineffective, biologically based resistance inducers represent a promising alternative for sustainable crop protection. This study evaluates the effectiveness of a liposomal bionanocomposite (BNC) based on a glycan–rhamnolipid complex as a seed treatment for enhancing the resistance of wheat (*Triticum aestivum* L.) to WSMV through modulation of antioxidant defenses and physiological responses. Wheat plants (cv. Zymoyarka) grown from BNC-treated seeds were assessed under mock- and virus-inoculated conditions. Analyses included viral titer dynamics (DAS-ELISA), indicators of redox homeostasis (H_2_O_2_, MDA), activities of antioxidant and stress-related enzymes (SOD, CAT, PAL), ethylene emission, chlorophyll content, and gas exchange parameters. Pre-sowing seed treatment with BNC resulted in a moderate reduction of WSMV accumulation, which was statistically significant at 14 days post-inoculation (16.02%). BNC treatment also mitigated oxidative stress by lowering MDA levels and modulating H_2_O_2_ dynamics compared to infected untreated plants. In virus-infected plants, it enhanced SOD and PAL activities, stabilized CAT responses, reduced ethylene overproduction, and partially restored chlorophyll content and photosynthetic performance. These results indicate that BNC-induced activation of antioxidant and stress-protective mechanisms improves the physiological resilience of wheat during WSMV infection and supports the potential of this biotechnological approach as a component of integrated crop protection against viral diseases.

**Key points:**

• *BNC stimulates antioxidant enzymes and modulates wheat redox homeostasis.*

• *Eco-friendly BNC offers a sustainable strategy for managing viral diseases.*

• *BNC induces systemic, pre-adaptive metabolic adjustments. *

**Graphical abstract:**

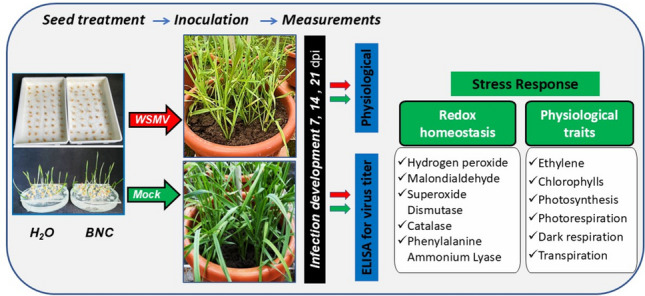

## Introduction

Crop production holds strategic importance for the sustainable development of the global economy. Cereal crops have served as not only a staple food for millennia but are also becoming renewable raw materials for industrial and energy applications (Pawlak and Kołodziejczak 2020). Wheat (*Triticum aestivum* L.) is one of the most important cereal crops due to its high ecological plasticity which allows cultivation across regions with well-developed agricultural systems (Bahar et al. 2020; Gao et al. 2021). The key task in achieving high grain yields is the realization of crop productivity potential under abiotic stress conditions (IPCC 2022). This challenge is further complicated by growing imbalances in the plant–pathogen–environment system, which cause substantial yield losses and poses a threat to future food security (FAO 2021).

The most distributed viruses affecting cereal crops and consistently detected in Ukrainian agroecosystems include wheat streak mosaic virus (WSMV; *Tritimovirus tritici*, genus *Tritimovirus*, family Potyviridae), High Plains wheat mosaic virus (HPWMoV; *Emaravirus tritici*, genus *Emaravirus*, family Fimoviridae), barley streak mosaic virus (BSMV; *Hordeivirus hordei*, genus *Hordeivirus*, family Virgaviridae), wheat dwarf virus (WDV; *Mastrevirus hordei*, genus *Mastrevirus*, family Geminiviridae), and barley yellow dwarf virus complex (B/CYDV, hereafter BYDVs) (Snihur et al. 2018, 2025). Many of these viruses pose a significant threat to cereal production, reducing grain yields and overall agricultural productivity, which has major commercial implications for the country and the global economy. Among them, WSMV is the most prevalent in Ukrainian wheat fields, capable of causing yield losses of up to 94% in susceptible wheat varieties and severely affecting chlorophyll content and yield components (Wosula et al. 2018).

Since chemical treatments are ineffective against viral diseases, integrated management strategies employing complex biopreparations are becoming increasingly important for enhancing wheat resistance to viruses. Complex biopreparations can modulate biochemical processes in plant cells, enhance antioxidant activity, and increase stress resistance to adverse factors (Mamenko and Yakymchuk 2019; Nyzhnyk et al. 2025). Biochemical defense systems play a central role in shaping stress resistance and plant adaptation to adverse environmental factors (Anzano et al. 2022; Hasanuzzaman et al. 2020). Under stress, plants undergo changes in gene expression, activate antioxidant enzymes, accumulate low-molecular-weight organic osmolytes, and increase ethylene synthesis and release (Segal and Wilson 2018; Singh et al. 2019; Kolupaev and Blume 2022). These adaptive responses are triggered by metabolic shifts and structural modifications in the cellular milieu, including the intensification of lipid peroxidation in biological membranes caused by excessive accumulation of reactive oxygen species (ROS) (Dumanović et al. 2021; Saed-Moucheshi et al. 2021; Lukan and Coll 2022).

There is a clear regulation of plant homeostasis that involves a multifactorial system responsible for both the production and neutralization of ROS. Two major systems of the plant organism operate simultaneously in maintaining this balance: the antioxidant system, which eliminates excess ROS, and the ROS-mediated signaling system, which activates protective responses (Laxa et al. 2019; He et al. 2020). Understanding the role of antioxidant systems involved in the stabilization of free-radical processes within cells and promoting the development of adaptive traits under environmental pressures is essential for developing strategies aimed at enhancing plant resilience. Thus, the search for effective and environmentally safe approaches for regulating plant redox homeostasis is critical for increasing stress tolerance.

The scientific hypothesis proposes that increasing plant tolerance to adverse factors can be achieved by activating endogenous antioxidant defenses through biological preparations. Such treatments enable targeted modulation of regulatory networks in plants and metabolic pathways, thereby increasing resistance to WSMV. An integrated approach to crop protection that employs liposomal bionanocomposites (BNCs) as bio-based resistance inducers can help realize the adaptive potential of plants and improve their stress tolerance in an environmentally compatible manner. Available toxicological and physiological assessments indicate that the BNC concentrations applied in our studies do not exert adverse effects on seedlings or selected nontarget organisms (Kyrychenko et al. 2025; Kyrychenko and Kovalenko 2025). Collectively, these results underscore the potential of the specific BNC formulation as a biologically based strategy for mitigating viral damage in wheat.

Previous studies demonstrated that glycan–glycolipid complexes (GGCs) composed of basidiomycete-derived glycans and bacterial rhamnolipids induce virus resistance in tobacco plants hypersensitive to tobacco mosaic virus (TMV) and protect soybean against soybean mosaic virus (SMV) under field conditions (Kovalenko et al. 2017, 2022, 2023). These GGCs also facilitated recovery of bean callus infected with bean common mosaic virus (BCMV) (Kovalenko et al. 2017). In the present study, we used one such glycan–rhamnolipid formulation designated hereafter as BNC, consisting of glucan from *Ganoderma adspersum* and rhamnolipids from *Pseudomonas* sp. PS-17, prepared according to Kovalenko et al. (2017). Thus, within this work, the term BNC denotes a specific bionanoformulation rather than a general class of glycan–glycolipid complexes.

The aim of this study was to evaluate the biotechnological potential of BNC seed treatment to enhance stress-protective responses and physiological performance of wheat under WSMV infection by assessing (i) oxidative processes, (ii) activation of key enzymatic systems, and (iii) photosynthetic activity, highlighting the application of microbial glycan–rhamnolipid complexes in a liposomal formulation as an innovative elicitor.

## Materials and methods

### BNC preparation

#### Rhamnolipid isolation (RhL)

Rhamnolipids (RhLs) were precipitated from the supernatant of a 14-day culture of *Pseudomonas* sp. PS-17 by acidification with 1 N HCl to pH 3.0, followed by extraction of the precipitate with Folch mixture (methanol:chloroform, 1:2, *v*/*v*), as described previously (Kovalenko et al. 2017). In this study, a RhL preparation additionally purified by heterophase extraction was also used (Kyrychenko et al. 2025).

#### Glucan extraction

**Fig. 1:**
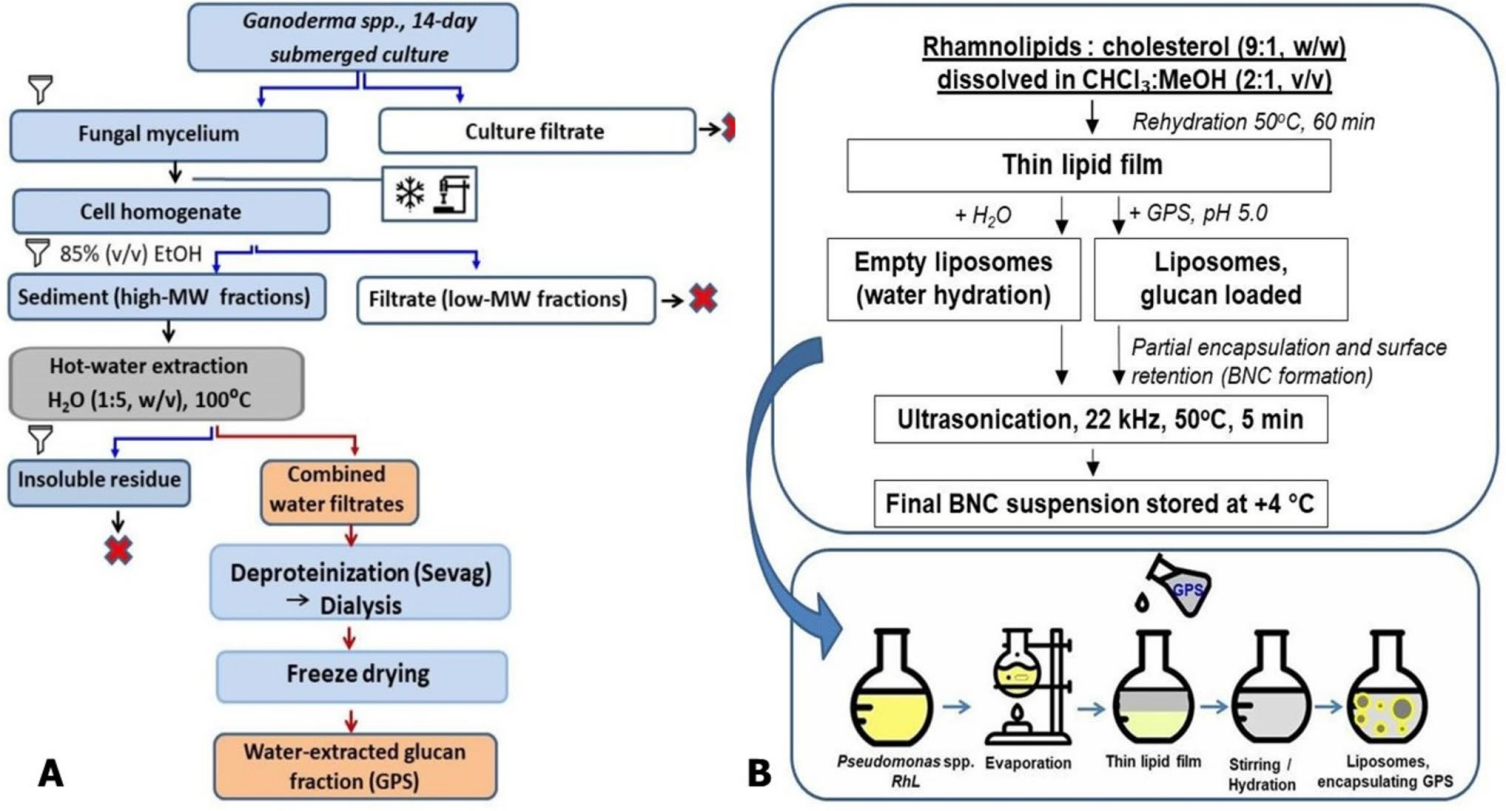
Schematic representation of BNC preparation. **A** Glucan extraction from *Ganoderma adspersum* mycelium, including removal of low-molecular-weight compounds, hot-water extraction, deproteinization, dialysis, and lyophilization to obtain the water-extracted glucan fraction (GPS). Red crosses (×) indicate discarded fractions. **B** Liposomal bionanocomposite formation via thin-film hydration of rhamnolipid–cholesterol mixture to produce empty or glucan-loaded liposomes, followed by sonication to reduce vesicle size and improve homogeneity

Glucan from *Ganoderma adspersum* (Schulzer) Donk was extracted from the mycelium and purified from impurities according to a modified method described previously (Kovalenko et al. 2010; Kovalenko and Wasser 2018). After 14 days of submerged cultivation, the mycelium was harvested by centrifugation (7000*g*, 20 min), washed with physiological saline, and freeze-dried. Low-molecular-weight impurities were removed from the ground lyophilizate by boiling the biomass in 85% (*v*/*v*) ethanol. The ethanol-insoluble residue (high-molecular-weight fraction) was extracted twice with distilled water at a ratio of 1:5 (*w*/*v*) at 100 °C for 3 h. The combined aqueous extracts were deproteinized using Sevag reagent (chloroform:isoamyl alcohol, 10:1, *v*/*v*), dialyzed against distilled water, and freeze-dried to obtain the water-extracted glucan fraction (GPS). The glucan extraction procedure is schematically presented in Fig. 1A.

#### BNC formation

BNCs were prepared by the thin-film hydration method according to standard protocols (Šturm and Ulrih 2021). RhLs were mixed with cholesterol at a mass ratio of 9:1 (*w*/*w*) and dissolved in chloroform:methanol (2:1, *v*/*v*). The organic solvent was evaporated under reduced pressure at 50 °C using a rotary evaporator to form a thin lipid film. The film was hydrated either with distilled water (to obtain empty liposomes) or with a GPS solution in phosphate buffer (pH 5.0) to obtain glucan-loaded liposomes. Hydration was carried out at 50 °C for 60 min with gentle stirring. The resulting suspension was sonicated (22 kHz, 50 °C, 5 min, cyclic mode) to reduce vesicle size and improve homogeneity. The liposome preparation procedure is illustrated in Fig. 1B.

### Brightfield microscopy of liposomes

Brightfield imaging of liposome samples was performed using a Meiji Techno MT4000 Series Biological Lab Microscope (Meiji Techno, Japan) equipped with an infinity-corrected optical system (focal length *F* = 200 mm). The microscope was fitted with 4 ×, 10 ×, S40 ×, and S100 × (oil immersion) objective lenses and a binocular or trinocular viewing head. Illumination was provided by a halogen or LED light source operating in transmitted brightfield mode.

Samples were examined at different magnifications to assess vesicle morphology and distribution. Representative micrographs were acquired at a total magnification of approximately 600 ×. Images were recorded using a digital microphotography system connected to the microscope.

### Plant material and pre-sowing treatment

The experiments were conducted using wheat (*Triticum aestivum* L.) cv. Zymoyarka. This mid-ripening cultivar was developed through individual plant selection from a spring wheat sample of German origin subjected to low temperatures during pre-winter sowing. The variety exhibits high genetic productivity potential and, under optimal mineral nutrition, yields 8.6–9.4 t/ha with superior grain quality (https://sops.gov.ua/ua/derzavnij-reestr). The originators of the variety are the Institute of Plant Physiology and Genetics of the National Academy of Sciences of Ukraine (NASU) and the V. M. Remeslo Myronivka Institute of Wheat of the Ukrainian Academy of Agrarian Sciences (NAAS).

Before sowing, wheat seeds were treated with BNC. The optimal concentration of BNC (50 mg L⁻^1^) was selected based on preliminary assays demonstrating antiviral efficacy without phytotoxicity (Kyrychenko and Kovalenko 2025). Seeds were soaked in BNC solution (experiment) or distilled water (control) at a ratio of 1:3 (*w*/*v*; 30 mL solution (or water) per 100 g seeds) for 8 h at room temperature (20–25 °C) and then air-dried for 24 h prior to sowing.

### Growth conditions and experimental design

Plants were cultivated in 10-kg pots filled with dark gray podzolic soil under controlled greenhouse conditions at the Institute of Plant Physiology and Genetics, NASU. Soil moisture was maintained at 70% of full water-holding capacity through regular controlled irrigation. Plants were grown under natural daylight. Each treatment consisted of six pots with 10 plants per pot. The following experimental variants were applied:

1. Control—untreated seeds, mock-inoculated;
2. BNC—BNC-treated seeds, mock-inoculated;
3. WSMV—untreated seeds, WSMV-inoculated;
4. BNC + WSMV—BNC-treated seeds, WSMV-inoculated.

Plant material for biochemical and physiological analyses and virus detection was collected at 7, 14, and 21 days post-inoculation (dpi).

### Virus isolate and plant inoculation

The WSMV isolate (WSMV-Vin-Tri; GenBank accession PV647599) was obtained from naturally infected wheat plants collected in the Vinnytsia region of Ukraine. The isolate was maintained on susceptible wheat cv. Zymoyarka under greenhouse conditions at the Plant Virology Laboratory, D.K. Zabolotny Institute of Microbiology and Virology, NASU. Leaves exhibiting characteristic WSMV symptoms were harvested and used for virus inoculum preparation. WSMV monoinfection of source plants was confirmed using double antibody sandwich enzyme-linked immunosorbent assay (DAS-ELISA).

Plants were inoculated at the tillering stage using the finger-rubbing method. Leaves were lightly dusted with carborundum, and 40 μL of inoculum was gently rubbed (three to four times to facilitate viral entry) onto each leaf (Ranabhat et al. 2022). For virus detection, the youngest fully expanded top leaf from each plant within a replicate was pooled into a composite sample for serological analysis.

### Serological testing

Leaf tissue (200 mg per replicate) was homogenized in extraction buffer (1:5, *w*/*v*) and tested for WSMV by DAS-ELISA using phytodiagnostic kits (Loewe Biochemica, Germany; 07048S/500). To verify the absence of mixed infections, additional DAS-ELISA tests were performed for other mosaic-causing cereal viruses prevalent in Ukraine using commercial polyclonal antisera: BSMV (Loewe Biochemica; 07004S/500), BMV (Loewe Biochemica; 07016S/500), and HPWMoV (Agdia, USA; SRA 17200/500). All procedures were performed according to the manufacturers’ protocols and the general methodological principles described by Clark and Adams (1977).

Samples were analyzed on the same ELISA plate to minimize inter-assay variability. Absorbance at 405 nm (*A*_405_) was measured using a Thermo Labsystems Opsys MR microplate reader (Thermo Fisher Scientific, USA) equipped with Dynex Revelation Quicklink software. Samples were considered WSMV-positive if *A*_405_ exceeded at least twice the negative control and was ≥ 0.2.

The reduction in viral titer (*R*, %) was calculated to evaluate the effect of BNC pre-sowing treatment on viral accumulation. This parameter was derived from DAS-ELISA absorbance values using the formula:

$$R (\%)=\frac{{A}_{\mathrm{WSMV}}- {A}_{\mathrm{BNC}+\mathrm{WSMV}} }{{A}_{\mathrm{WSMV}}} \times 100$$

where *A*_WSMV_ and *A*_BNC+WSMV_ represent the mean *A*_405_ values for the WSMV and BNC + WSMV variants, respectively, at each time point (*n* = 6 biological replicates).

### Determination of hydrogen peroxide (H_2_O_2_)

Leaf tissue (500 mg per replicate) was homogenized in chilled 5% (*w*/*v*) trichloroacetic acid (1:3, *w*/*v*). The supernatant was obtained by centrifugation at 14,000 rpm for 5 min at 4 °C. H_2_O_2_ concentration was determined spectrophotometrically at 480 nm based on the color reaction with potassium thiocyanate and calculated using a calibration curve (Sagisaka 1976). The results are expressed as µmol g of fresh weight (FW).

### Intensity of lipid peroxidation (LPO)

Malondialdehyde (MDA), the end product of LPO, was quantified using a UV-1900 scanning dual-beam spectrophotometer (Shimadzu, Japan) (Heath and Paker 1968). Leaf tissue (500 mg per replicate) was homogenized in 3 mL distilled water, followed by the addition of 3 mL of 0.1% trichloroacetic acid (TCA) and repeated homogenization. Two 2-mL aliquots of homogenate were prepared: One was mixed with 2 mL of 20% TCA (control), and the other with 2 mL of 0.5% thiobarbituric acid in a 20% TCA (test). Absorbance of supernatants was measured at *λ* = 532 nm and corrected for nonspecific absorption at 600 nm. MDA content was calculated using the molar extinction coefficient *ε* = 1.55 × 10^5^ cm^−1^ M^−1^ and expressed as nmol of MDA per g of FW.

### Extraction and determination of superoxide dismutase (SOD) activity

To obtain the enzyme extract, a weight of plant material (0.5 g) was ground in a mortar with 4 mL of 50 mM phosphate buffer (pH 7.5), which contained 2 mM EDTA, 1 mM PMSF, 5 mM β-mercaptoethanol, and 1% polyvinylpyrrolidone. The homogenate was centrifuged at 10,000 rpm for 20 min at 4ºC. The activity of SOD in the supernatant (EC 1.15.1.1) was determined by its ability to inhibit the photochemical reduction of nitroblue tetrazolium (Raychauhuri and Deng 2000). The reaction proceeded for 15 min at a light intensity of 70 μmol quanta/(m^2^ s) illumination by fluorescent lamps with a power of 15 W. The optical density was measured at 560 nm. The results are presented in units of enzyme activity (U) per mg of protein in the supernatant.

### Extraction and determination of catalase activity (CAT)

To obtain the enzyme extract, a portion of the plant material was triturated (ratio 1:2) with a cooled 0.5 M Tris–HCl buffer (pH 7.8) containing 5 mM β-mercaptoethanol and 0.1% polyvinylpyrrolidone. The homogenate was centrifuged at 10,000 rpm (4ºC) for 20 min. The supernatant was taken and assayed for CAT activity (EC 1.11.1.6.) by the development of a color reaction with ammonium molybdate; the concentration was measured at a wavelength of 410 nm according to Doliba et al. 2010). The results are presented in units of enzyme activity (U) per mg of protein in the supernatant.

### Phenylalanine ammonia-lyase (PAL)

To obtain the enzyme extract, the plant material was homogenized with a 0.2 M solution of borate buffer (pH 8.8) in a ratio of 1:2 (*w*/*v*), which contained 1 mM EDTA, 5 mM β-mercaptoethanol, and 1% polyvinylpyrrolidone (*w*/*v*). The homogenate was centrifuged at 12,000 rpm for 20 min at 4ºC. The supernatant was used to determine PAL activity (EC 4.3.1.5) spectrophotometrically at 290 nm by the formation of trans-cinnamic acid in 0.1 M borate buffer (pH 8.8) in the presence of 50 mM L-phenylalanine (Zucker 1969). The reaction mixture was incubated for 1 h at 40 °C. The results are presented as units of enzyme activity (U) per mg of protein in the supernatant.

### Ethylene emission measurement

One whole wheat leaf was placed in 75 mL glass vials, which were immediately sealed and left in the dark for 24 h (Guzmán and Ecker 1990). After incubation, the gas mixture containing ethylene was analyzed on an Agilent GC system 6850 gas chromatograph (USA). The volume of the analyzed gas mixture sample was 1 mL. Pure ethylene (Sigma-Aldrich, USA) was used as a standard. The amount of ethylene released from the incubated sample was expressed as µmol C_2_H_4_ per plant per hour.

### Determination of chlorophyll content

The wheat flag leaves (0,5 g) was covered with dimethylsulfoxide and placed in a water bath at a temperature of + 63 °C for 3 h to ensure complete extraction. The obtained extract was diluted in dimethylsulfoxide (ratio of 1:9). The optical density of the obtained solution was determined at wavelengths of 649 and 665 nm (Wellburn 1994).

### Gas exchange rate measurement

The photosynthesis rate was measured under controlled conditions using an open gas exchange system equipped with an infrared gas analyzer (GIAM-5M) operating in differential mode. The middle sections of two intact leaves from the main shoot were placed in a temperature-controlled chamber maintained at 25 °C and illuminated with a TA-11 50 W LED spotlight (color temperature 5200 K). The light intensity at the chamber level was 1500 μmol·m^−2^·s^−1^) of photosynthetically active radiation. Conditioned atmospheric air, with a humidity of 9.5–10.0 mbar, flowed through the chamber at a flow rate of 1 L·min^−1^. The transpiration rate was measured using an EGM-5 gas analyzer (PP Systems, USA) and calculated based on the difference in air humidity between the inlet and outlet of the leaf chamber. CO_2_ concentration was monitored at the chamber inlet. Gas exchange parameters were calculated according to Busch et al. 2024).

Measurements were performed at a single representative time point (14 dpi), chosen to capture the characteristic physiological responses of wheat under WSMV infection and BNC seed treatment. This approach minimized experimental variability while remaining feasible under greenhouse conditions.

### Statistical data analysis

Statistical analyses were performed using STATISTICA ver. 13.3 software package (StatSoft Inc. 2024). All parameters were compared using two methods.

Statistical analyses were performed using the STATISTICA software package, version 13.3 (StatSoft Inc., 2024). Two complementary approaches were applied, selected based on the type of data and their distribution.

*Physiological and biochemical parameters* were analyzed using the nonparametric Kruskal–Wallis test. This test was chosen because the data did not fully meet the assumptions of normality and homogeneity of variance required for parametric tests, and it allows comparison across four independent groups: untreated controls (no BNC, mock-inoculated), BNC (BNC-treated seeds, mock-inoculated), WSMV (untreated seeds, inoculated), and BNC + WSMV (BNC-treated seeds, inoculated). Each group included six biological replicates per time point (7, 14, and 21 dpi). Results are presented in the figures and table, with letters indicating significant differences between groups (*p* < 0.05).

*DAS-ELISA data* (viral accumulation) were assessed for normality and homogeneity of variance using the Shapiro–Wilk and Levene’s tests, which confirmed approximately normal distribution and similar variances across the two groups (WSMV and BNC + WSMV). Therefore, a parametric two-tailed Student *t*-test for independent samples was applied, which is appropriate for normally distributed continuous data, even with a relatively small sample size (*n* = 6). Mean absorbance values (*A*_405_) and standard deviations (SD) were calculated for each time point (7, 14, and 21 dpi), and viral titer reduction (*R*, %) was derived from these values as described in the “Serological testing” section. Differences were considered statistically significant at *p* ≤ 0.05.

This approach ensures that the statistical analyses are tailored to the characteristics of the data and the experimental design, providing robust and reliable evaluation of treatment effects.

### Formulation and biotechnological considerations

Liposomal BNCs were prepared to ensure stability, reproducibility, and scalability. Liposomal delivery protects bioactive components, enables controlled release, and enhances targeted action, representing a biotechnological innovation rather than a technical detail.

## Results

### BNC characterization

**Fig. 2:**
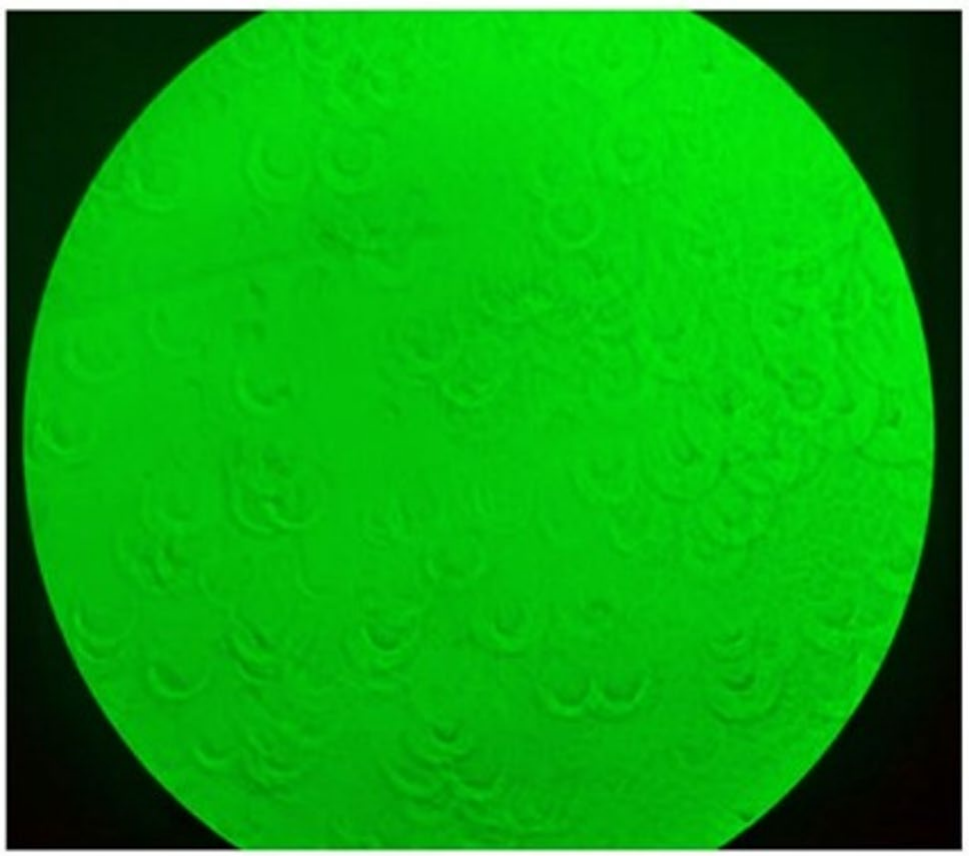
Brightfield microscopy of empty liposomes. Representative light microscopy image of liposomes at 600 × magnification obtained using a Meiji Techno MT4000 Series Biological Lab Microscope (Meiji Techno, Japan). Microphotograph provided by O. G. Kovalenko

BNC morphology was examined by light microscopy (Olympus BX53, phase-contrast mode) to confirm vesicular structure, glucan encapsulation (partial internal loading combined with surface retention due to intermolecular interactions), and the absence of large aggregates (Fig. 2). According to light microscopy data, liposome size ranged from 0.1 to 0.5 µm.

### WSMV infection symptoms and viral accumulation dynamics

**Fig. 3:**
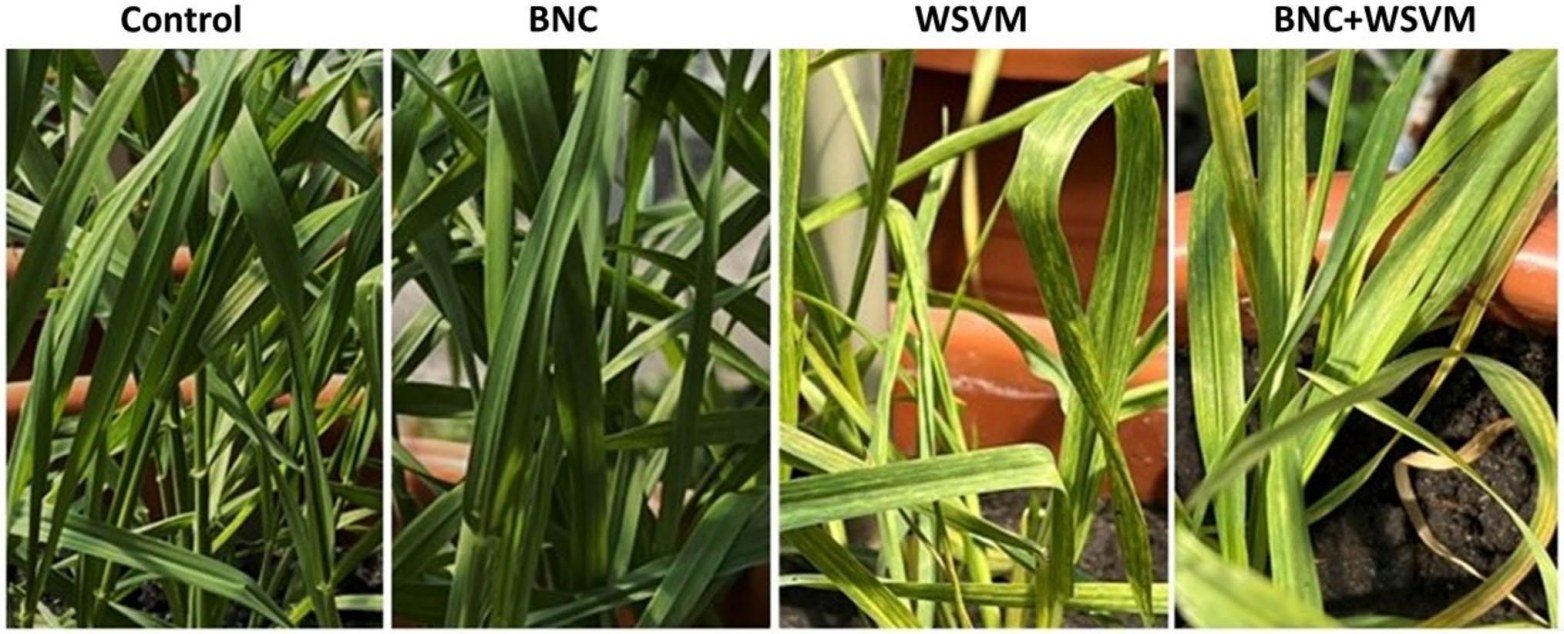
Visual assessment of wheat plants at 14 dpi under different experimental treatments: control, BNC, WSMV, and BNC + WSMV. Symptoms include chlorotic streaks and mosaic in infected plants

Visual inspection of plants at 14 dpi revealed characteristic WSMV symptoms, including chlorotic streaks, mosaic patterns on the leaves, yellowing, and slight leaf deformation. Plants of the BNC + WSMV variant showed moderately reduced symptom severity compared with the WSMV variant (Fig. 3). Although chlorotic streaks and mosaic patterns were still visible, their intensity was noticeably lower, which was consistent with the DAS-ELISA data.

Table 1 presents the dynamics of WSMV titer accumulation in wheat samples collected from all experimental variants at 7, 14, and 21 days post-inoculation. Viral infection was confirmed exclusively in the WSMV and BNC + WSMV treatments. In both variants, the highest viral titers (ELISA absorbance values) were recorded at 7 dpi, followed by a decline at 14 and 21 dpi. At 14 dpi, corresponding to the peak phase of systemic virus spread, plants of the BNC + WSMV variant showed a statistically significant but modest 16.02% reduction in viral titer compared with infected plants without pre-sowing treatment (*p* = 0.0115, Student’s *t*-test, *n* = 6). No significant differences (*p* > 0.05) between plants grown from BNC-treated and untreated seeds were detected at 7 dpi (2.01) or 21 dpi (2.09%), indicating a transient moderating effect on viral accumulation during the active infection phase.

**Table 1:** WSMV titer accumulation in wheat during infection (DAS-ELISA)*

Time point	(Mean ± SD)		Reduction in viral titer (%)	*p* value (*t*-test, *n* = 6)
	WSMV	BNC + WSMV		
7 dpi	0.943 ± 0.041	0.924 ± 0.010	2.01	0.2821
14 dpi	0.788 ± 0.058	0.661 ± 0.082	16.02	0.0115**
21 dpi	0.766 ± 0.041	0.750 ± 0.049	2.09	0.5530

*DAS-ELISA controls: k + (positive control): 1.057; k−1 (negative control 1, sap of virus-free plants): 0.021; k−2 (negative control 2, buffer): 0.011

**p < 0.05

### Plant redox homeostasis

**Fig. 4:**
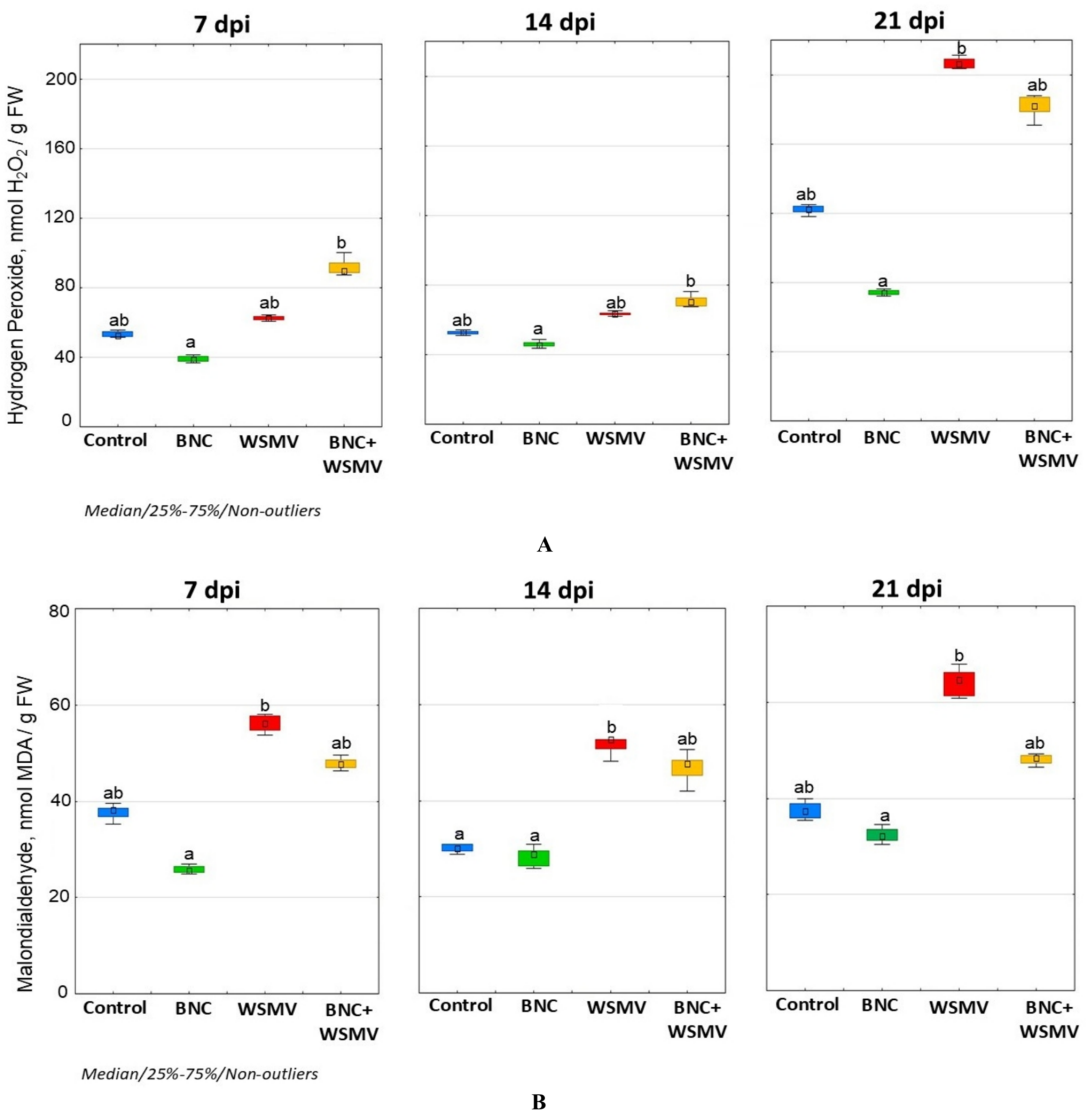
Effect of BNC seed treatment on hydrogen peroxide (**A**) and malondialdehyde (**B**) contents in leaves. Letters indicate significant differences between variants (*p* < 0.05; Kruskal–Wallis test). Experimental treatments untreated controls (mock-inoculated); BNC (seed-treated, mock-inoculated); WSMV (untreated, inoculated); BNC + WSMV (seed-treated, inoculated)

WSMV infection triggered pronounced H_2_O_2_ overproduction in wheat, peaking on day 21 post-infection at 179.3% above the control level (Fig. 4A). Concurrently, an intensification of lipoperoxidation processes occurred due to excessive accumulation of MDA in wheat leaves under stress, increasing by 117.4% at 7 dpi and 98.1% at 21 dpi (Fig. 4B).

Under optimal growth conditions, BNC seed treatment significantly reduced H_2_O_2_ and MDA levels compared to BNC-untreated controls (Fig. 4A, B). At 7 dpi, the highest H_2_O_2_ generation (91.6 nmol/g FW) occurred in the BNC + WSMV variant, which was 72.5% above the control value (Fig. 4A). At 21 dpi, H_2_O_2_ levels in BNC-treated plants were 48.7% higher than in untreated control plants. WSMV infection also significantly increased MDA content in BNC-treated leaves, which was 48% higher than in the control (Fig. 4B), although values remained lower than in untreated infected plants.

### Enzyme activities

**Fig. 5:**
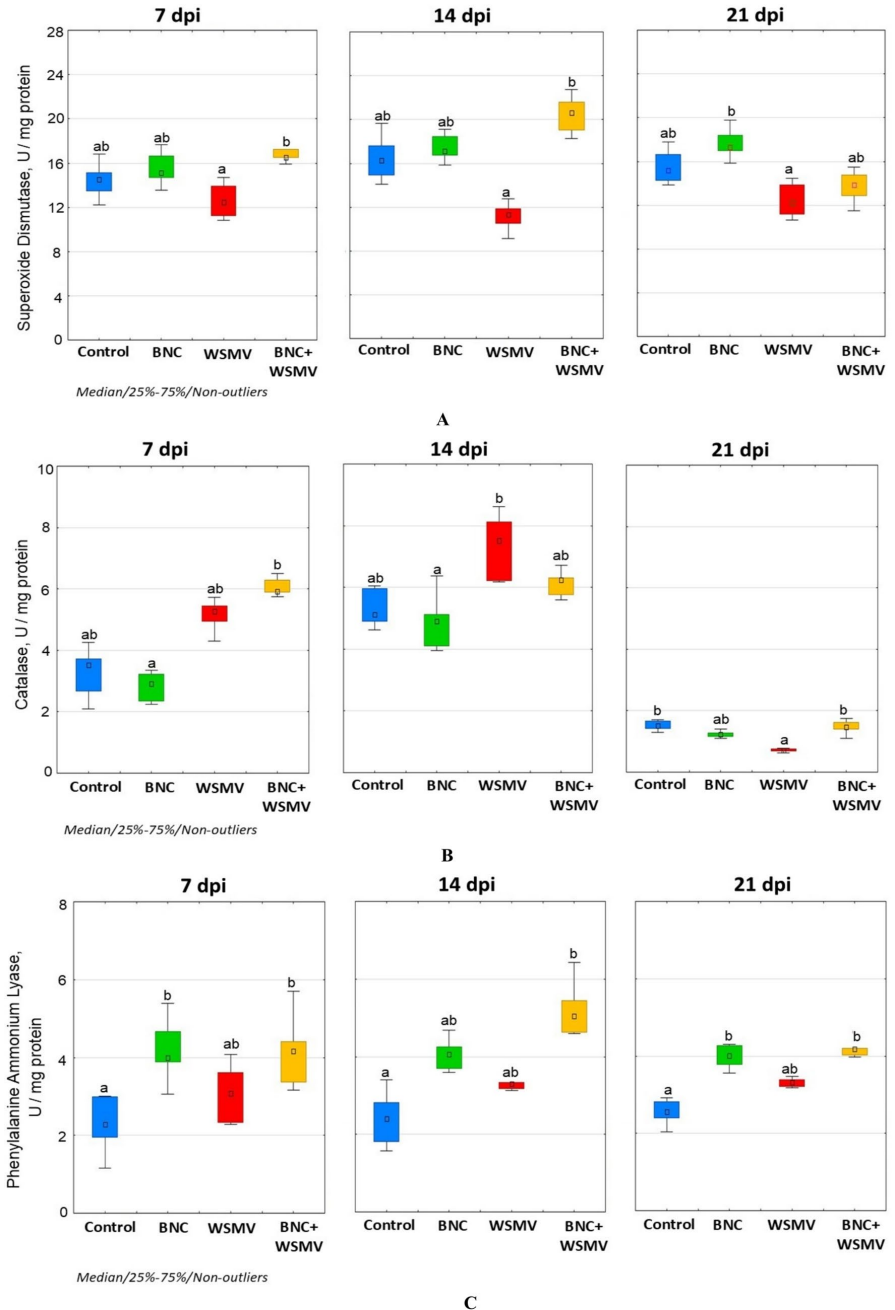
Effect of BNC seed treatment on superoxide dismutase (**A**), catalase (**B**), and phenylalanine ammonium lyase (**C**) activities. Other details as in Fig. 4

WSMV-infected plants showed the lowest SOD activity compared to other experimental variants. In particular, at 21 dpi, enzyme activity was 20% lower than the control (Fig. 5A). Under optimal growth conditions, BNC seed treatment induced a statistically significant increase in SOD activity by 7.5–13.5% compared to the control (Fig. 5A). BNC treatment of WSMV-infected plants caused an increase in SOD activity by 17.8% at 7 dpi and 23.8% at 14 dpi relative to the control (Fig. 3A). At 21 dpi, activity slightly decreased and was 11.9% below the control.

CAT activity under WSMV infection showed unstable dynamics, with an increase at 14 dpi (7.3 U/mg protein) and a sharp decrease at 21 dpi (0.7 U/mg protein) (Fig. 5B). BNC seed treatment reduced the overall level of CAT activity in wheat leaves under optimal growth conditions (Fig. 5B). In infected plants treated with BNC, the highest level of CAT activity was recorded on the seventh day of infection development, where it was 86.5% higher than the control. At 21 dpi, no significant differences remained between BNC + WSMV and control variants (Fig. 5B).

During WSMV infection, an increase in PAL activity was recorded by 30.1–39.1% relative to the control (Fig. 5C). Under optimal conditions, BNC treatment also induced a significant increase in PAL activity. However, the maximum PAL activity occurred in BNC + WSMV plants at 14 dpi, reaching 117.9% above control.

### Physiological responses of plants

**Fig. 6:**
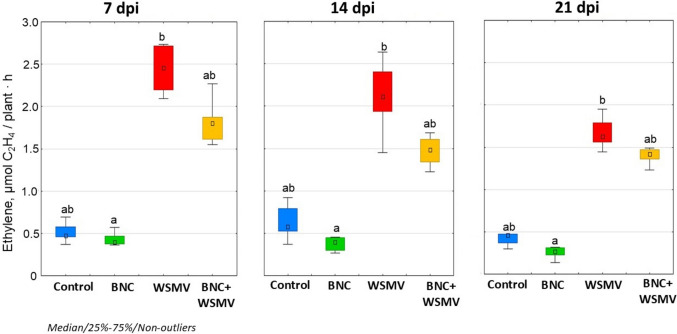
Effect of BNC seed treatment on ethylene emission. Other details as in Fig. 4

Ethylene production was highest in virus-infected plants. At 21 dpi, ethylene release reached 2.4 μmol/plant h, which was 5.5-fold higher than the control (Fig. 6). BNC seed treatment under optimal conditions reduced hormone synthesis, reaching the lowest level (0.2 μmol/plant h) at 21 dpi (Fig. 6). This value was approximately twofold lower than the control. During infection, BNC + WSMV plants showed statistically significant ethylene release (from 1.4 to 1.8 μmol/plant h), although values remained lower than in untreated infected plants.

**Fig. 7:**
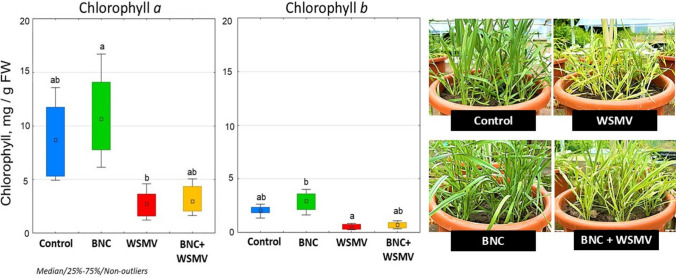
Effect of BNC seed treatment on chlorophyll content (mg/g FW; combined 7 + 14 dpi). Other details as in Fig. 4

WSMV reduced chlorophyll a and b to the lowest levels among all variants (2.7 and 0.5 mg/g FW, respectively) (Fig. 7). BNC treatment under optimal conditions caused the highest values of chlorophyll content: 11.1 mg/g FW for chlorophyll a and 2.8 mg/g FW for chlorophyll b. Under infection, pigment content decreased in BNC + WSMV plants but remained higher than in infected plants grown from untreated seeds (Fig. 7).

Among all treatments, the lowest photosynthesis rate was recorded in virus-infected plants [10.66 μmol CO_2_/(m^2^ s)], which was 20% below the control (Table 2). BNC treatment during disease development (BNC + WSMV variant) resulted in a nonsignificant decrease in photosynthesis [12.21 μmol CO_2_/(m^2^ s)], which was 11.1% lower than the control level (Table 2). At the same time, the highest photorespiration rates were detected in both variants with BNC treatment, with an increase of 34.8% compared to untreated control (Table 2). Dark respiration and transpiration displayed no statistically significant differences among the variants.

**Table 2:** Gas exchange in leaves of wheat plants*

Treatments	Net assimilation rate μmol CO_2_/(m^2^ s)	Photorespiration μmol CO_2_/(m^2^ s)	Dark respiration μmol CO_2_/(m^2^ s)	Transpiration mmol H_2_O/(m^2^ s)
Control	13.73 ± 0.41^a^	1.81 ± 0.05^a^	1.30 ± 0.04^b^	2.13 ± 0.06^a^
BNC	13.05 ± 0.39^a^	2.27 ± 0.07^ab^	1.13 ± 0.03^a^	1.88 ± 0.06^b^
WSMV	10.66 ± 0.32^b^	1.98 ± 0.06^a^	1.49 ± 0.04^b^	1.80 ± 0.05^b^
BNC + WSMV	12.21 ± 0.37^ab^	2.44 ± 0.07^b^	1.47 ± 0.04^b^	1.91 ± 0.06^b^

*Temperature 25 °C, illuminance 1500 μmol/(m^2^ s); these parameters were measured on 14 dpi; other details as in Fig. 4

## Discussion

The dynamics of oxidative processes and the activity of key enzyme complexes in wheat were analyzed during the progression of WSMV infection. Leaf samples from virus-infected plants were collected at 7, 14, and 21 days post-inoculation, corresponding respectively to the onset of systemic virus movement (6–10 dpi) and the transition to stationary phase of viral development (14–28 dpi) (Ranabhat et al. 2022). Table 1 represents viral titer measurement data and provides a quantitative basis for interpreting the associated physiological and biochemical responses.

Visual manifestations of WSMV infection corresponded well with viral accumulation patterns. Plants grown from BNC-treated seeds exhibited attenuated symptom development, particularly at 14 dpi, when ELISA confirmed a modest, transient reduction. These results indicate that BNC seed priming mitigates WSMV-induced symptoms and partially preserves physiological homeostasis and stress responses, which may limit virus-induced damage despite ongoing infection. Although the reduction in viral titer was modest and time-limited, the observed activation of antioxidant defenses (SOD, PAL, CAT stabilization) and preservation of photosynthetic function align with mechanisms of induced systemic tolerance and induced systemic resistance in other plant-virus systems. In such cases, even partial viral suppression, combined with enhanced redox homeostasis and maintained physiological performance, can mitigate disease impact and contribute to yield protection under field conditions (Paudel and Sanfaçon 2018; Yu et al. 2022; Ghanaim et al. 2025).

WSMV triggered substantial change in redox metabolism, as evidenced by elevated H_2_O_2_ and MDA levels throughout the infection period (Fig. 4A, B). Their gradual increase to 21 dpi indicates severe oxidative stress and confirms the high sensitivity of wheat to WSMV. Notably, BNC pretreatment modified these dynamics, and although H_2_O_2_ levels in treated plants temporarily exceeded those of untreated infected plants at 7 and 14 dpi, they declined by 21 dpi, falling below the levels observed in infected controls (Fig. 4A). The results obtained suggest that early H_2_O_2_ accumulation in BNC-treated plants reflects a signaling function rather than uncontrolled oxidative damage.

Such an interpretation aligns with the well-established concept that ROS, including H_2_O_2_, are among rapidly induced defense signals during biotic and abiotic stress (Dumanović et al. 2021). Acting as a signaling molecule, H_2_O_2_ can activate kinase and stimulate the expression of defense-related genes important for stress adaptation (Hasanuzzaman et al. 2020; Lukan and Coll 2022). The observed MDA accumulation rates support this interpretation. Lipid peroxidation was markedly increased in WSMV-infected plants, while BNC pretreatment significantly reduced LPO intensity compared with untreated infected plants, as indicated by lower MDA concentrations (Fig. 4B). This reduction is consistent with the early signaling role of H_2_O_2_, which may trigger timely the activation of plant antioxidant defenses. Since cell membranes serve as key sites for ROS perception and downstream signal transduction (Noctor et al. 2018), the moderate LPO observed in BNC-treated plants likely reflects improved regulation of redox homeostasis.

An important role in the perception of ROS signals is played by cell membranes, where the majority of enzymatic complexes involved in the initiation of calcium-, lipoxygenase-, and phosphatide-oxalate-dependent signaling pathways are located (He et al. 2020). Consequently, lipid peroxidation products inevitably participate in the regulation of homeostatic systems in plant cells. ROS-mediated restructuring of cellular metabolic processes, including lipid peroxidation, represents one of the earliest components of stress establishment, while timely activation of antioxidant systems is a crucial determinant that provides the plant organism with enhanced tolerance (Segal and Wilson 2018; Laxa et al. 2019; Kolupaev and Blume 2022). These mechanisms were particularly evident in our experiments, where BNC-mediated early modulation of H_2_O_2_ correlated with reduced MDA accumulation (48% above controls in BNC + WSMV and 117.4% in untreated WSMV at 7 dpi), indicating a prepared adaptive response similar to defense mechanisms characteristic of tolerant wheat genotypes under WSMV pressure in Ukrainian field conditions (Mishchenko et al. 2021).

The key antioxidant enzyme protecting plants from oxidative damage is superoxide dismutase, which catalyzes the dismutation reaction to neutralize the overproduction of superoxide radicals under stress (Saed-Moucheshi et al. 2021). However, the efficiency of SOD depends on downstream peroxide-scavenging systems, primarily catalase and various peroxidases, which remove the H_2_O_2_ generated during dismutation (Laxa et al. 2019). WSMV disrupted this balance, suppressing SOD activity (20% below controls at 21 dpi) and causing unstable CAT dynamics peaking at 14 dpi and then declining by 21 dpi (Fig. 5A, B). Such responses indicate impaired redox regulation in untreated infected plants, consistent with progressive antioxidant depletion. Increased SOD activity often observed under prolonged stress may reflect the activation of latent isoforms and (or) de novo synthesis of the enzyme. However, once oxidative pressure exceeds a critical threshold, SOD activity typically declines due to enzyme inactivation or degradation decreases (Wang et al. 2018; Shorning et al. 2000; Saed-Moucheshi et al. 2021). BNC pretreatment counteracted these disruptions during virus spread. SOD activity was higher at 7 and 14 dpi in BNC-treated infected plants compared to untreated infected plants (Fig. 5A). Although SOD activity declined by 21 dpi in both groups, the decrease was less pronounced in BNC + WSMV plants, suggesting delayed oxidative exhaustion.

Catalase responded differently. In BNC-treated plants, CAT activity exhibited an early increase at 7 dpi (86.5% above controls), followed by normalization at 21 dpi, no longer differing significantly from the control (Fig. 5B). These dynamics indicate more efficient H_2_O_2_ detoxification and stabilization of redox homeostasis, consistent with enhanced peroxisomal activity observed in tolerant wheat genotypes under WSMV infection (Mishchenko et al. 2021).

Hydrogen peroxide also upregulates PAL, a key enzyme of the phenylpropanoid pathway responsible for producing phenolics, phytoalexins, lignin precursors, and other metabolites necessary for plant development and defense (Zhang and Liu 2015; Chen et al. 2017). PAL induction through ethylene- and jasmonate-dependent signaling is considered a key marker of systemic acquired resistance (Zhang and Liu 2015). WSMV caused only moderate PAL increase (Fig. 5C), reflecting modest defense activation and limited engagement of phenylpropanoid-mediated defense. In contrast, BNC pretreatment markedly enhanced PAL activity, particularly at 14 dpi (117.9% above control levels). These data support the conclusion that under nonstress conditions, BNC reduced H_2_O_2_ and MDA levels and increased SOD and PAL activities, suggesting that BNC initiates low-intensity metabolic shifts that may facilitate subsequent stress adaptation.

Intermediate and final products of lipid peroxidation, including MDA, can modify membranes by interacting with membrane proteins and phospholipids. Such changes may contribute to ethylene induction through ROS-dependent crosstalk (Noctor et al. 2018; He et al. 2020), thus altering the properties of both these components and the membrane and further affecting cellular signaling. Ethylene regulates many developmental and stress acclimation processes across nearly all stages of plant ontogenesis (Chang 2016). Its production typically rises shortly after exposure to a stressor and precedes visible symptom development (Wang et al. 2002). Although ethylene biosynthesis is activated by the generation of ROS and is essential for specific stress-protective responses, at elevated levels ethylene accelerates leaf senescence (Ali et al. 2025).

WSMV markedly increased ethylene release (Fig. 6), indicating metabolic disruption and premature aging. BNC pretreatment minimized this effect, reducing basal ethylene levels (twofold below controls) and suppressing stress-induced peaks, thus promoting protective rather than degenerative responses (Wang et al. 2002). Higher ethylene levels in BNC + WSMV plants during early infection likely participate in activating early stress-protective reactions of plants in response to damage by a phytopathogen. Ethylene can coordinate the upregulation of antioxidant enzymes (SOD, CAT, PAL) and redistribution of energy resources, including enhanced photorespiration (Table 2). In addition, ethylene is also required for signal transduction, metabolic reprogramming, and the initiation of general and specific adaptation cascades (Wang et al. 2002).

Although photorespiration is an energy-consuming process that uses ATP, formed as a result of the electron transport chain (ETC) functioning in chloroplasts, it plays a protective role when various stress factors affect the photosynthetic apparatus, which inhibit its activity (Broncano et al. 2023). Viral infection also belongs to the latter, since the photosynthetic apparatus needs to be provided with nitrogen-containing compounds, primarily amino acids for the synthesis of structural and enzyme proteins. Under viral infection, competition for such compounds arises between the photosynthetic apparatus and the virus, since the latter switches nitrogen metabolism to the synthesis of its own proteins. As a result of the inhibition of CO_2_ assimilation due to the lack of enzyme proteins, a risk of over-reduction of ETC components arises. In this case, electrons transferring directly to molecular oxygen (Mehler reaction) may occur with the formation of dangerous ROS that damage photosynthetic structures. At the same time, photorespiration acts as an alternative electron sink from ETC, which prevents its over-reduction and the formation of excess ROS. This is indirectly evidenced by the decrease in MDA content (Fig. 4) and the increase in chlorophyll content (Fig. 7) in BNC-treated plants. In addition, the products of photorespiration are important amino acids—glycine and serine, that is, it also participates in the plant nitrogen metabolism.

In wheat WSMV severely suppressed photosynthesis (Table 2) and chlorophyll content (Fig. 7), both indicators of disrupted redox balance and core metabolic pathways disintegration that often precede accelerated plant aging. BNC pretreatment preserved pigment content and attenuated the decline in photosynthetic activity without altering dark respiration or transpiration rates (Table 2). These results suggest that BNC redirects metabolic energy toward defense while maintaining the efficiency of photosynthetic processes under infection pressure. Such a targeted action, combined with the well-documented biodegradability and low ecotoxicity of rhamnolipid-based formulations compared to synthetic surfactants (Hogan et al. 2019; Lavanya 2024), positions BNC as a promising environmentally compatible strategy for viral disease management in cereal crops.

Nanocarrier-based delivery systems, such as nanoliposomes encapsulating quercetin and other flavonoids, have been explored for enhancing antiviral efficacy in plants by targeted gene silencing or improved uptake (Wang et al. 2022). However, the use of rhamnolipid-based liposomes loaded with fungal glycans remains largely unexplored. Previous studies from our group demonstrated that glycan–glycolipid complexes (GGCs), composed of basidiomycete-derived glycans and bacterial rhamnolipids, effectively reduced the infectivity of TMV, SMV, and common bean mosaic virus and induced resistance in both hypersensitive and susceptible plant models (Kovalenko et al. 2017, 2022, 2023). Optimization of rhamnolipid production and characterization of its extracellular metabolites further confirmed their antiviral activity (Kyrychenko et al. 2025). The present study extends this concept by applying a BNC to wheat infected with WSMV, a widespread and economically significant pathogen in Ukrainian agroecosystems. Unlike many elicitor delivery systems that primarily function as passive delivery vehicles, the proposed BNC formulation combines the inherent antiviral properties of both components—rhamnolipids and fungal glucans—with the biotechnological advantage of liposomal delivery. This dual-function design enables improved penetration into wheat tissues and coordinated activation of antioxidant defenses (SOD, CAT, PAL), stabilization of redox balance, modulation of ethylene signaling, and maintenance of photosynthetic tolerance during WSMV infection. Although the precise molecular mechanism remains to be elucidated, this work demonstrates the feasibility of engineering microbially derived, bio-based liposomal compositions that simultaneously function as delivery systems and biologically active elicitors. This represents a conceptual shift from carrier-based nanotechnology toward multifunctional bioactive nanoformulations for plant viral protection.

Despite the promising results of this study, several limitations should be considered. Experiments were conducted under controlled greenhouse conditions, which may not fully capture the complexity of field environments. Additionally, the study was limited to a single wheat cultivar, and responses may vary across other varieties. Nevertheless, the applied BNC concentrations were shown to have no toxic effects on seedlings or selected nontarget organisms, supporting their environmental safety and suitability for crop protection applications.

## Conclusions

BNC seed treatment enhances the antioxidant and stress-protective responses of wheat to WSMV infection by stimulating hydrogen peroxide–dependent signaling and activating key components of the antioxidant defense network, including SOD, CAT, and PAL. Such coordinated enzymatic activation attenuates lipid peroxidation and contributes to the restoration of redox homeostasis. BNC also modulates ethylene production and supports the functioning of the photosynthetic apparatus in WSMV-infected plants.

An increase in photorespiration observed in BNC-treated plants may contribute to maintaining redox balance and preventing excessive reactive oxygen species accumulation under stress conditions. However, as photorespiration is an energy-demanding process, this response likely represents a physiological trade-off between protective redox regulation and metabolic cost.

Collectively, BNC induces pre-adaptive metabolic adjustments, strengthens antioxidant capacity, reduces WSMV-associated oxidative damage, and improves physiological performance under infection. These protective effects on redox homeostasis, ethylene regulation, and photosynthetic function are more pronounced and consistent than the observed influence on viral accumulation, indicating that BNC primarily enhances physiological tolerance to WSMV rather than exerting strong direct antiviral activity.

## Data Availability

The datasets generated during and/or analyzed during the current study are available from the corresponding author on reason able request.
